# Rib Fragility Fractures and Chest Wall Hematoma After Cardiopulmonary Resuscitation Training: A Case Report

**DOI:** 10.7759/cureus.47998

**Published:** 2023-10-30

**Authors:** Troy Nguyen, Amy Yu

**Affiliations:** 1 College of Medicine, Lake Erie College of Osteopathic Medicine, Erie, USA; 2 Department of Radiology, Tulane University School of Medicine, New Orleans, USA

**Keywords:** cardiopulmonary resuscitation, spontaneous fractures, osteoporosis, osteopenia, chest compression, cpr, rib fractures, fragility fractures

## Abstract

Fragility fractures commonly manifest as complications in individuals with diminished bone mineral density and other risk factors. The hip, vertebral body, and wrist are the most documented locations of fragility fractures among patients with osteoporosis or osteopenia. This report presents a rare case of multiple fragility fractures of the right ribs accompanied by an adjacent right chest wall hematoma in an otherwise healthy 60-year-old woman after participating in cardiopulmonary resuscitation (CPR) training. Upon further workup, a diagnosis of osteopenia was established. This report aims to underscore a potential complication in those performing CPR and outline the clinicoradiological presentations, diagnostic workup, and treatment of fragility fractures in patients with no history of prior underlying skeletal conditions or malignancy.

## Introduction

Rib fractures, sternal fractures, and lung contusions are well-documented complications in patients undergoing cardiopulmonary resuscitation (CPR) [1]. However, there is limited data on the complications arising in CPR performers and a lack of CPR guidelines on modified methods or health criteria for individuals at high risk of injuries like fractures. Fractures are categorized into traumatic and non-traumatic etiologies. The latter, interchangeably referred to as spontaneous, low-energy, pathologic, or fragility fractures, manifest in individuals with various underlying conditions, thus necessitating prompt diagnostic workup. A rib fracture incurred non-traumatically after administering CPR, whether on a real person or a mannequin, falls within the domain of fragility fractures.

This is a case report of a 60-year-old woman who sustained multiple rib fragility fractures and a chest wall hematoma following participation in CPR training. Subsequent workup confirmed the diagnosis of osteopenia. This report aims to highlight the clinicoradiological presentations of rib fragility fractures as well as the typical diagnostic workup and management in a patient without prior malignancy or underlying skeletal conditions. It also underscores the need for caution among osteopenic or osteoporotic patients when engaging in strenuous activities such as chest compressions.

## Case presentation

A 60-year-old woman presented to the outpatient clinic with the chief complaint of right shoulder and upper back pain. She reported that the pain began after a CPR class a few days prior. Bruising later developed over the area of pain. An image captured on her phone revealed a small area of bruising on the right posterior flank at the start of the pain. Over a few days, the pain worsened, and the bruised area gradually expanded over the entire right posterior flank, likely over the region of the right trapezius and latissimus dorsi muscles. The patient reported receiving a subacromial steroid injection by an orthopedic specialist within days following the CPR class. She was additionally prescribed cyclobenzaprine hydrochloride and meloxicam, which she denied taking due to concerns about worsening bruising. She admitted to taking acetaminophen and leftover hydrocodone. The remaining review of systems and past medical history at this time were negative for prior trauma, anticoagulant consumption, or diagnosis of cancer or any underlying bone condition. Her surgical, familial, and social histories were non-contributory to the current presentation.

**Figure 1:**
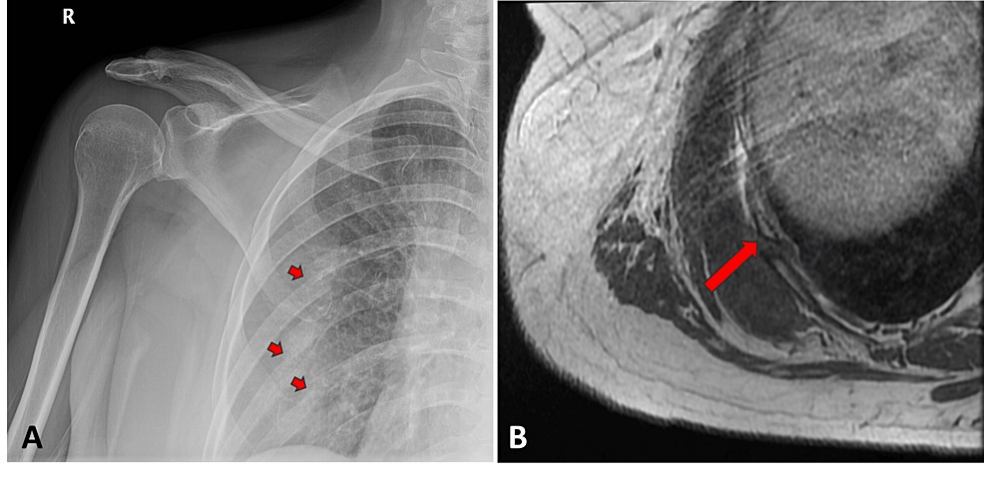
(A) Frontal view of the right shoulder radiograph showed multiple consecutive fractures of the right posterior sixth through eighth ribs (arrows). (B) Axial T1-weighted MRI of the chest demonstrated several subacute mildly displaced right posterolateral rib fractures (arrow) with callus formation and adjacent soft tissue edema.

**Figure 2:**
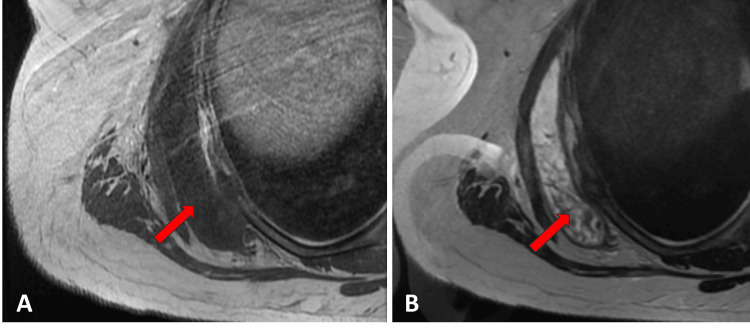
Axial MR of the chest demonstrated a well-defined collection measuring 14.4 x 10.2 x 1.8 cm (CC x AP x TR) between the right serratus anterior muscles and right external intercostal muscles exhibiting T1 hypointensity (A) and T2 hyperintensity and containing layering intermediate-to-low signal material within the dependent portion (B), likely representing a chest wall hematoma (arrows). CC: craniocaudal; AP: anteroposterior; TR: transverse

**Figure 3:**
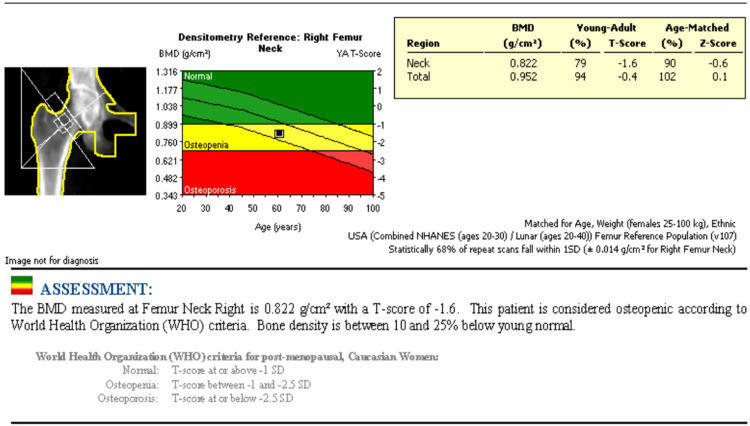
DEXA scan revealed the BMD of the right femoral neck was 0.822 g/cm2 with a T-score of -1.6 after being matched for age, gender, weight, and ethnicity, confirming the diagnosis of osteopenia. DEXA: dual x-ray absorptiometry; BMD: bone mineral density

The physical exam demonstrated an area of tenderness and muscular distension palpated over the right posterior flank, possibly related to the right trapezius and/or latissimus dorsi muscle, accompanied by significant bruising. The remaining physical exam was normal. The patient's comprehensive blood cell counts with differential were all within normal limits, which suggested against hematological malignancy. X-ray of the right shoulder in the frontal view revealed multiple mildly displaced rib fractures (Figure 1A). A magnetic resonance imaging (MRI) study of the chest and abdomen without and with contrast was obtained for further musculoskeletal investigation. The MRI confirmed several subacute mildly displaced right posterolateral rib fractures with callus formation and adjacent soft tissue edema (Figure 1B). MRI also depicted a well-defined collection between the right serratus anterior muscles and external intercostal muscles measuring 14.4 x 10.2 x 1.8 cm (craniocaudal x anteroposterior x transverse), likely representing a chest wall hematoma (Figures 2A, 2B). A subsequent mammogram yielded negative results for suspected breast lesions (BI-RADS 2). A dual x-ray absorptiometry (DEXA) scan was completed and revealed a T-score of -1.6, confirming osteopenia (Figure 3).

The patient was counseled to adopt a bone-healthy lifestyle, incorporating weight training routines and a diverse diet. Vitamin D supplementation and bisphosphonate therapy were initiated. A routine DEXA scan was also recommended every two years. The patient has not had a follow-up visit since the encounter for the diagnostic workup, although no symptom exacerbation was reported.

## Discussion

Rib fractures are categorized as traumatic or nontraumatic, with thoracic trauma being the most common cause of all rib fractures [2]. Nontraumatic rib fractures, on the other hand, have been associated with various risk factors, e.g., osteopenia, osteoporosis, metastatic breast cancer, osteosarcoma, aromatase inhibitor therapy, radiation to the chest wall, prolonged use of bisphosphonates, and advanced age over 83 years [3]. Among individuals with such risk factors, elevated intrathoracic pressure due to forceful coughing, sneezing, or heavy lifting can precipitate spontaneous rib fractures [2]. For example, forceful coughing prompts the contraction of inspiratory and expiratory muscles, inducing alterations in intrapleural pressure. Subsequently, a counter-directional force is exerted upon the ribs, leading to fractures in regions of decreased structural integrity [2]. Rib fragility fractures can also be genetically driven, e.g., overexpressed BMP3 gene or hypocalciuric hypercalcemia [4,5]. The use of systemic steroids, particularly oral forms, as a treatment for inflammatory or autoimmune conditions increases the susceptibility to fractures. The increased osteoporosis risk corresponds with higher cumulative steroid exposure [6].

Radiographs can identify fragility fractures but have many limitations, successfully detecting only 20-38% of such fractures [7]. Conversely, computed tomography (CT) scans can reveal fracture lines, changes in osseous density, and eventual fracture callus formation in fragility fractures. Nonetheless, their sensitivity has been reported at only 60-75% [8]. CT scans prove helpful in showing cortical and trabecular destruction within neoplastic lesions and, thus, are recommended for fragility fractures in patients with recurrent or metastatic breast cancer [9]. Alternatively, MRI is highly sensitive in diagnosing fragility fractures, although its specificity is limited when distinguishing them from neoplastic lesions. Typical MRI findings of fragility fractures include bone marrow edema, which is best visualized on fluid-sensitive, fat-saturated sequences, and fracture line, which is best seen on T1-weighted sequences [9]. When no additional bone lesions are observed beyond fragility fractures on MRI, age-appropriate cancer screenings in high-risk patients who have not undergone routine screenings are recommended, as demonstrated by a mammogram in our case of a postmenopausal woman. Pertinent tumor biomarkers may also be considered, for instance, CA-125, 15-3, and CEA, in a patient with a history of recurrent breast cancer [3].­

DEXA scans offer precise evaluation of mineralized bone content in the skeletal system, significantly enhancing the detection of osteopenia and osteoporosis. According to the DEXA evaluation criteria by the World Health Organization (WHO), bone mineral density (BMD) T-scores falling between -1 and -2.5 at the spine or hip indicate osteopenia, whereas T-scores < -2.5 indicate osteoporosis [10]. DEXA scans are recommended for all women aged 65 and above and men aged 70 and above, regardless of osteoporotic signs [11]. Furthermore, DEXA scans are also indicated for women below 65 and men below 70 who exhibit osteoporosis risk factors, such as ongoing smoking, height reduction, thoracic kyphosis, and estrogen deficiency [10]. Additionally, the WHO has introduced the Fracture Risk Assessment (FRAX) tool to better identify those at high risk of osteoporotic fractures. This assessment computes fracture risk by considering significant fracture determinants, including age, gender, body weight, body mass index, fracture history, parental hip fracture, current smoking, excessive alcohol consumption, rheumatoid arthritis, glucocorticoid usage, and other secondary osteoporosis forms [11].

Over 80% of postmenopausal women with fractures have T-scores > -2.5. Since the number of postmenopausal women in the osteopenic range is much higher than those in the osteoporotic range, 82% of fragility fractures occur in the osteopenic range, aligning with our presented case [12]. A patient with the first nonvertebral fracture stands twice the risk of a subsequent fracture. The risk for a subsequent fracture is even quadrupled after a vertebral fracture. Nearly half of all subsequent fractures occur within three to five years following the initial fracture [13]. Thus, prompt intervention is imperative to prevent recurring fractures, minimizing morbidity and mortality [13]. Most guidelines addressing decreased BMD underscore fragility fractures as the definitive criterion for initiating pharmacologic therapy [11,14]. For individuals with osteopenia without fragility fractures or low FRAX scores (10-year risks < 20% for all osteoporotic fractures and < 3% for hip fractures), first-line treatments involve adopting a bone-healthy lifestyle, including optimal nutrition, regular exercise, smoking cessation, and vitamin D and calcium supplements if needed [14]. Antiresorptive treatments such as bisphosphonates or denosumab serve as the primary therapies for all cases of osteopenia associated with fractures or elevated FRAX scores (10-year risks ≥ 20% for all osteoporotic fractures and ≥ 3% for hip fractures). Hormone replacement and selective estrogen receptor modulators (SERMs) can be considered in premenopausal women with contraindications to antiresorptive treatments [14].

CPR is essential to basic life support (BLS) or advanced cardiovascular life support (ACLS). However, it can be physically demanding, especially for those with low BMD, leading to a higher fracture risk. Limited data exist on CPR guidelines for high-risk rescuers. Modified CPR methods can be considered, like mechanical devices or increased core and body weight engagement to reduce arm strain. Additionally, rotating rescuers can alleviate fatigue and strain on only one individual. CPR rescuers at high risk should consult a medical professional or certified CPR instructor before performing CPR.

## Conclusions

This case report highlights the susceptibility to fragility fractures among osteopenic individuals, particularly when participating in physically demanding activities such as CPR. While hip, vertebral body, and wrist fractures are the most frequent fractures associated with osteoporosis, the systemic impact of osteopenia on the skeleton translates to an elevated risk of fractures in various skeletal structures, including less common sites like ribs. Consequently, there is a necessity for enhanced health criteria for CPR rescuers. Moreover, prompt diagnostic evaluations and a multidisciplinary approach to managing fragility fractures are essential in reducing the likelihood of recurrent fractures, thus minimizing subsequent morbidity and mortality.
